# Analysis of the time course and prognostic factors determining toxicity due to infused fluorouracil

**DOI:** 10.1038/sj.bjc.6600917

**Published:** 2003-05-13

**Authors:** N C Tebbutt, A R Norman, D Cunningham, M Allen, I Chau, J Oates, M Hill

**Affiliations:** 1 Gastrointestinal Unit, Royal Marsden Hospital, London and Surrey, UK; 2 Department of Computing and Information, Royal Marsden Hospital, London and Surrey, UK

**Keywords:** fluorouracil, adverse effects, time course, prognostic factors

## Abstract

This study used a prospectively managed clinical database in order to identify 1470 patients with gastrointestinal cancers receiving protracted venous infusion (PVI) fluorouracil (5FU). It aimed to determine the time course of toxicity due to PVI 5FU and to analyse factors predicting toxicity. The initial development of stomatitis occurred more rapidly than diarrhoea or palmar plantar erythema (PPE). The percentage of patients with National Cancer Institute Common Toxicity Criteria (CTC) grade 2 or worse PPE peaked at 9% between weeks 8 and 17, whereas this peak occurred earlier for stomatitis and diarrhoea. The development of CTC grade 1 toxicity in the first 28 days after commencement of chemotherapy was classified as early grade 1 toxicity. Multivariate Cox regression analysis showed that female sex, better performance status, elevated bilirubin, early grade 1 PPE and early grade 1 diarrhoea were independent prognostic factors for the development of CTC grade 2 or worse PPE (*P*<0.01). Female sex, increased age, elevated alanine transaminase and urea and early grade 1 PPE were significant independent prognostic factors for the development of CTC grade 2 or worse stomatitis (*P*<0.01). Early CTC grade 1 diarrhoea predicted CTC grade 2 or worse diarrhoea (*P*<0.01). Older, female patients with good performance status and impaired liver and renal function who develop early grade 1 PPE alone or in combination with diarrhoea are at highest risk of subsequently developing grade 2 or worse PPE or stomatitis during treatment with PVI 5FU. Reduction of infused 5FU dose should be considered for these patients. Such an approach could both reduce severe toxicity owing to chemotherapy and minimise treatment delays, and should be evaluated prospectively.

Fluorouracil (5FU)-based chemotherapy is used for the adjuvant and palliative treatment of a variety of different tumour types, particularly for gastrointestinal cancers. A recent meta-analysis demonstrated that the efficacy of 5FU was dependent on its schedule of administration. Infused schedules of 5FU resulted in superior response rates with a small but statistically significant prolongation of survival in advanced colorectal cancer compared with bolus schedules (Meta-analysis Group In Cancer, 1998a). In addition, the toxicity profile of infused 5FU differs from bolus schedules. Infused 5FU results in similar incidences of diarrhoea and stomatitis, but causes a lower incidence of haematological toxicity and a higher incidence of palmar–plantar erythema (PPE). Thus, diarrhoea, stomatitis and PPE form the most frequent and the dose-limiting toxicities for infused 5FU (Meta-analysis Group In Cancer, 1998b).

The development of chemotherapy-induced toxicity has an adverse impact on quality of life and in the most severe cases may cause death during treatment. The management of toxicities owing to chemotherapy are generally made on an empirical basis only after the development of dose-limiting side effects and generally involve interruption of chemotherapy and dose reduction in order to prevent future episodes. This study aimed to analyse the principal dose-limiting toxicities of infused 5FU therapy observed in a large cohort of patients treated at a single centre. It aimed to identify clinical, laboratory and temporal factors, which could be combined in order to identify those patients who were at greatest risk of toxicity.

## PATIENTS AND METHODS

### Patients and toxicity assessment

The Gastrointestinal Unit (GI unit) at the Royal Marsden Hospital (RMH) has maintained a prospective database of all patients receiving chemotherapy. This database contains details of baseline demographic data, haematological and biochemical parameters, chemotherapy administration, tumour response to chemotherapy and survival and toxicity data graded according to the National Cancer Institute Common Toxicity Criteria version 1 (Macdonald *et al*, 1995). Toxicities were recorded on a weekly basis for patients receiving infused 5FU regimens. Diarrhoea, PPE and stomatitis were selected as the toxicities to study, as these are the most frequent and dose-limiting side-effects of infused 5FU.

### Chemotherapy

Patients were eligible for this analysis if they had received protracted venous infusion (PVI) 5FU 300 mg m^−2^ day^−1^ alone or PVI 5FU 300 mg m^−2^ day^−1^ and bolus Mitomycin C (MMC) 7 mg/m^2^ q 6 weekly. MMC is associated with a mild and predominantly haematological toxicity profile; consequently in patients receiving MMC with PVI 5FU, the toxicities of diarrhoea, PPE and stomatitis were considered to most likely result from 5FU (DeVita *et al*, 1993). Patients receiving any other chemotherapy or concurrent radiotherapy were excluded from this analysis, as these other treatments would be likely to affect diarrhoea or stomatitis.

Treatment with PVI 5FU with or without MMC was continued up to 24 weeks, unless patients developed progressive disease or excessive toxicity.

### Empirical management of toxicity

Nonhaematological toxicities developing during PVI 5FU therapy were managed uniformly as follows. Grade 1 stomatitis and diarrhoea were treated with oral sucralfate and codeine phosphate, respectively, and grade 1 PPE was treated with oral pyridoxine. No dose reduction or delay of 5FU was made for grade 1 toxicities. Grade 2 or worse nonhaematological toxicities led to cessation of 5FU treatment until resolution of toxicity followed by a dose reduction of 5FU except for PPE developing for the first time after 10 weeks of therapy, where treatment was initially recommended at the original starting dose. Otherwise, grade 2 toxicities led to a 50 mg m^−2^ dose reduction, grade 3 toxicities to 100 mg m^−2^ dose reduction and grade 4 toxicities to 150 mg m^−2^ dose reduction. Therefore, for this analysis dose-limiting toxicities were defined as grade ⩾2 toxicities as this severity of toxicity would usually result in a dose reduction.

### Statistical methods

Time to the development of toxicity was calculated using the inverted methods of Kaplan and Meier (1958). Multivariate Cox regression analysis was used to generate multivariate proportional hazards models based on times to events as stated in the results. Factors included in the analyses were clinical factors (age, sex, performance status, type of primary tumour, presence of metastatic disease, chemotherapy arm), biochemical parameters (liver and renal function) and temporal factors. Hazard ratios associated with continuous variables other than age were not proportional across the full range of the variable. Therefore, continuous variables other than age were grouped as categorical values based on the median value of the variable. *P*-values of less than 0.01 were considered significant.

## RESULTS

### Patient demographics and overall toxicity

Between January 1994 and January 2001, 1470 patients with gastrointestinal cancer received treatment using infused 5FU at the Royal Marsden Hospital (RMH). The baseline demographics of these patients are shown in Table 1

**Table 1:** Baseline demographics of patients treated with PVI 5FU

	**Number**	**Percentage**
*Total*	1470	100.0
PVI 5FU alone	861	58.6
PVI 5FU plus MMC	609	41.4
Male	833	56.7
Female	637	43.3
Performance status 0–1	1081	73.6
Performance status 2–3	364	24.8
Unknown performance status	25	1.7
Age: median (range)	64 (24–86)	
Palliative treatment	1131	76.9
Adjuvant treatment	339	23.1
		
*Primary tumour*		
Oesophago-gastric	172	11.7
Colorectal	955	65.0
Pancreas	245	16.7
Unknown primary	98	6.7

. The percentage of patients who developed diarrhoea, PPE or stomatitis at any time during treatment is shown in Table 2

**Table 2:** Percentage of patients with varying grades of toxicity

	**Grade of toxicity**				
	**0**	**1**	**2**	**3**	**4**
Diarrhoea	58.4	23.2	13.1	3.6	1.7
PPE	37.1	28.0	28.0	6.9	
Stomatitis	51.3	21.5	21.7	4.3	1.2

. However, these data provide no information relating to when the toxicity occurred or which patients were at greatest risk of developing toxicity.

### Time course of initial development of toxicity

Figure 1A-3A

**Figure 1:**
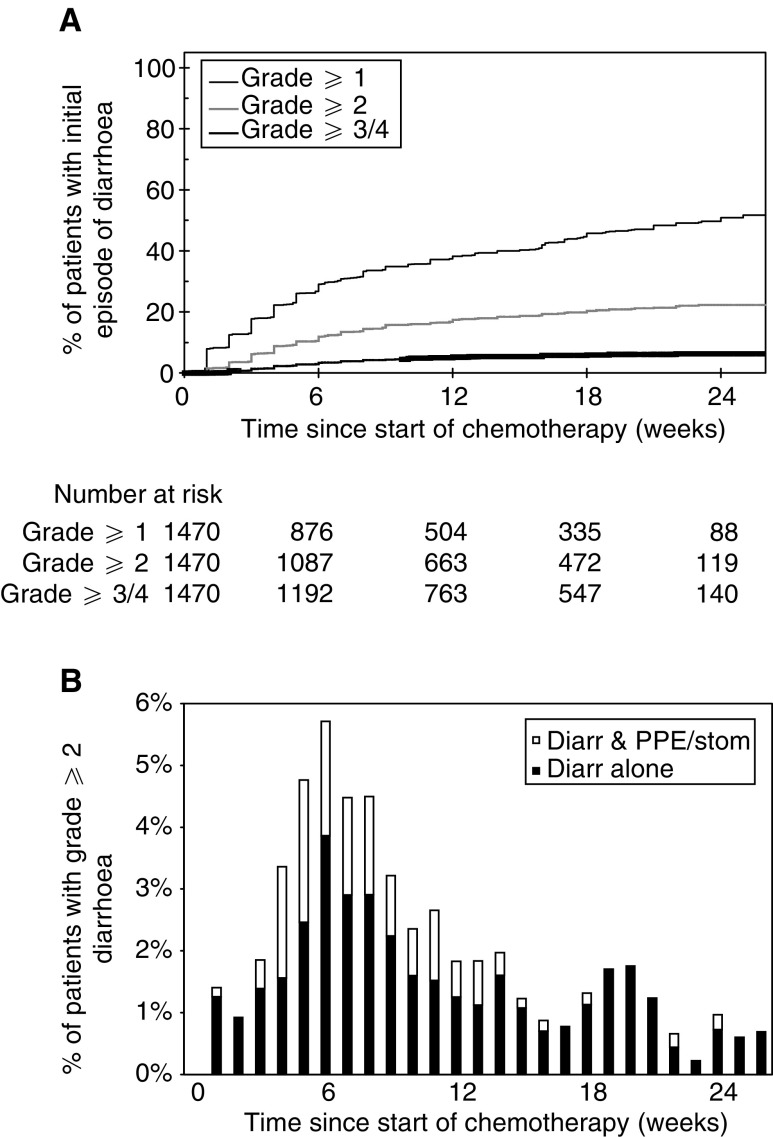
(**A**) Time until initial development of varying grades of diarrhoea. (**B**) Proportion of patients receiving PVI 5FU with grade 2 or worse diarrhoea. Closed columns indicate patients with only grade ⩾2 diarrhoea, whereas open columns indicate patients with grade ⩾2 diarrhoea and grade ⩾2 stomatitis and/or PPE.

**Figure 2:**
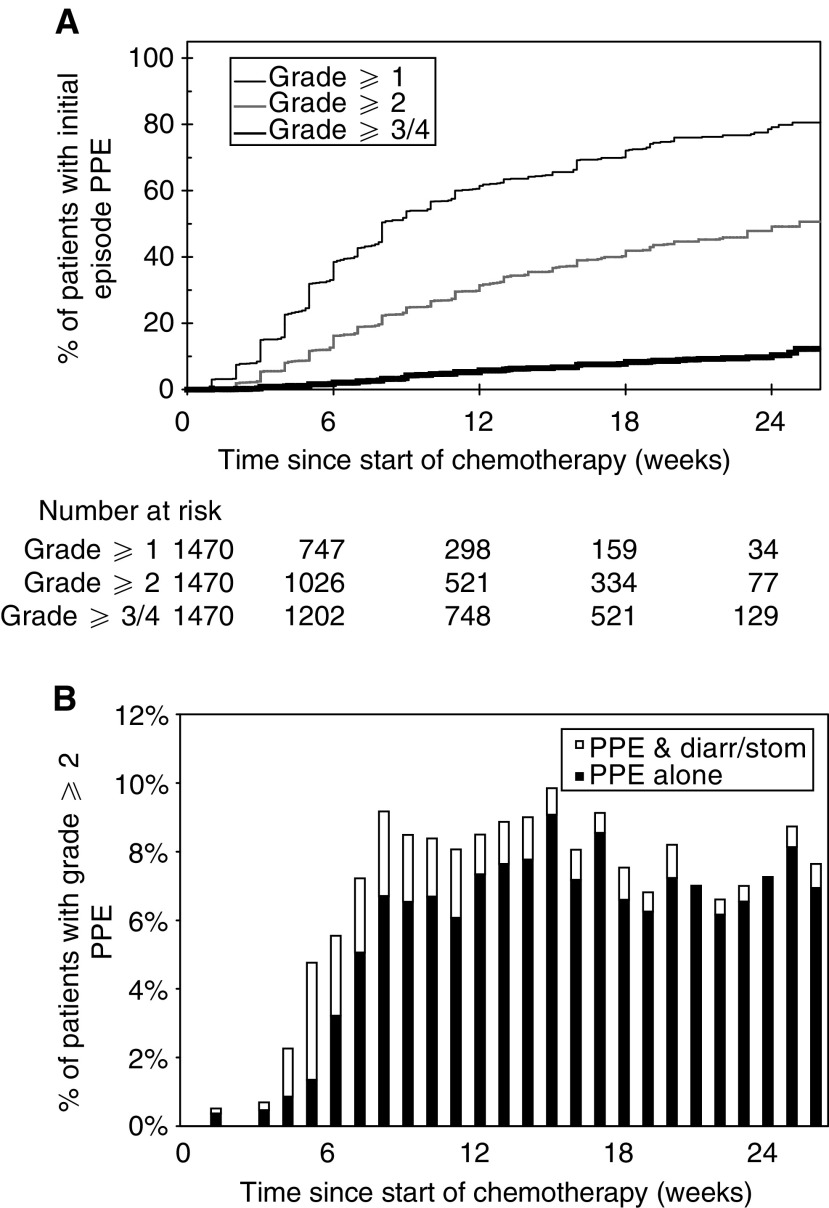
(**A**) Time until initial development of varying grades of PPE. (**B**) Proportion of patients receiving PVI 5FU with grade 2 or worse PPE. Closed columns indicate patients with only grade ⩾2 PPE, whereas open columns indicate patients with grade ⩾2 PPE and grade ⩾2 diarrhoea and/or stomatitis.

**Figure 3:**
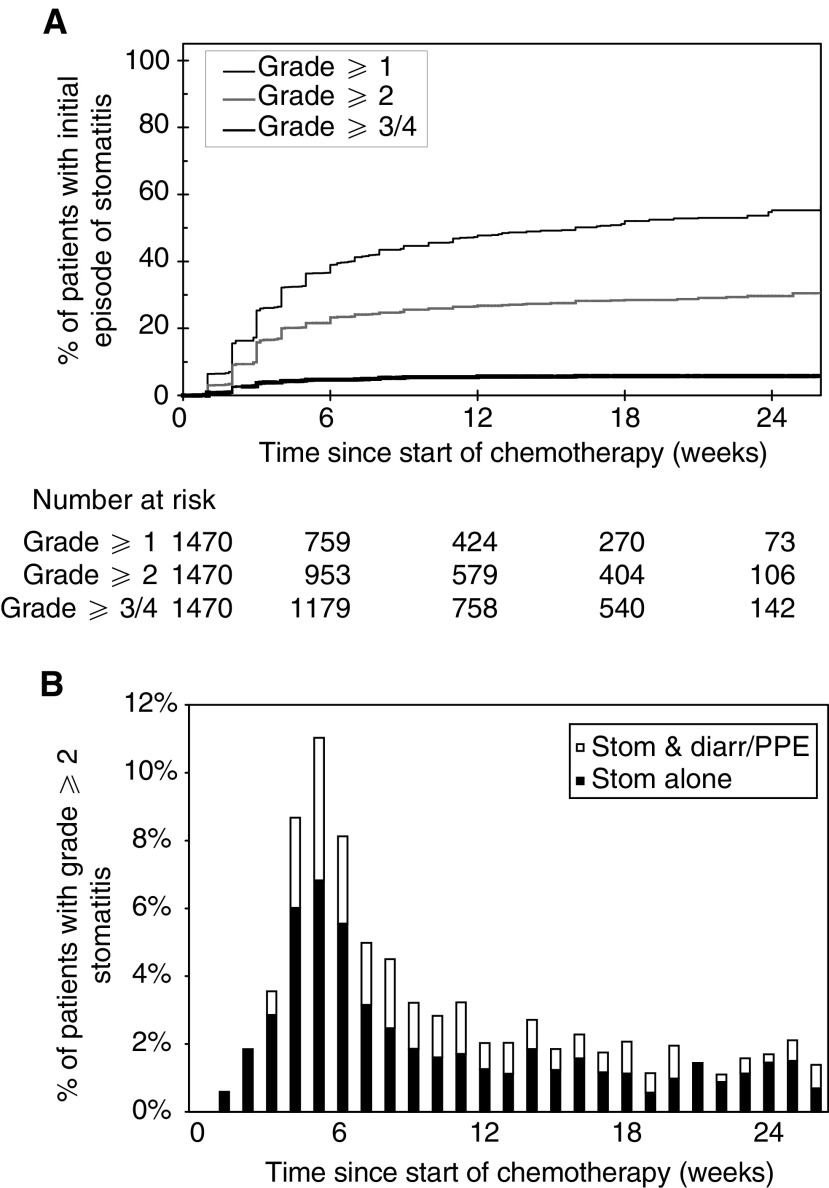
(**A**) Time until initial development of varying grades of stomatitis. (**B**) Proportion of patients receiving PVI 5FU with grade 2 or worse stomatitis. Closed columns indicate patients with only grade ⩾2 stomatitis, whereas open columns indicate patients with grade ⩾2 stomatitis and grade ⩾2 diarrhoea and/or PPE.

demonstrate the time to the first development of varying grades of diarrhoea, PPE and stomatitis for patients receiving chemotherapy. The baseline was defined as the date of commencement of chemotherapy and the events were the grades of toxicities encountered. The denominator at each time point was the number of patients receiving chemotherapy at that time and patients were censored at the chemotherapy stop date if no toxicity was encountered. The stepped profile of these curves reflects the weekly collection of toxicity data. Small numbers of patients continued treatment beyond 24 weeks as a result of previous treatment interruptions due to prior toxicities. The time course for the development of these three individual toxicities varied according to the toxicity. Thus, the initial development of stomatitis frequently occurred soon after commencement of chemotherapy, whereas the initial development of diarrhoea and PPE was more gradual with many cases occurring for the first time towards the end of therapy.

### Proportion of patients with dose-limiting toxicities during treatment

The time course of grade 2 or worse toxicities was studied in further detail, as this severity usually required modification of chemotherapy dose. The proportion of patients with grade 2 or worse diarrhoea, PPE and stomatitis was therefore determined during each week of chemotherapy. These results depict both initial as well as subsequent episodes of grade 2 or worse toxicities for all patients who were continuing to receive chemotherapy. The denominator at each time point was the number of patients receiving chemotherapy at that time. The percentage of patients with grade 2 or worse diarrhoea peaked at about 4.5% between weeks 5 and 8. Approximately 30% of patients with grade 2 or worse diarrhoea also had another grade 2 or worse toxicity (Figure 1B). The percentage of patients with grade 2 or worse PPE peaked at about 9% between weeks 8 and 17, and the majority of these patients had grade 2 or worse PPE alone without any other toxicity (Figure 2B). The percentage of patients with grade 2 or worse stomatitis peaked between weeks 4 and 6, with approximately 30% of patients also suffering another grade 2 or worse toxicity (Figure 3B).

### Identification of early grade 1 toxicities

While the development of more severe toxicities led to a dose delay or dose reduction, the development of individual grade 1 toxicities alone did not mandate any dose modification. The risk of developing a worse grade toxicity after the development of an initial grade 1 toxicity was analysed in more detail, in order to assess whether the time of onset of grade 1 toxicity had any prognostic significance for the subsequent development of more severe toxicity. Univariate Cox regression analysis demonstrated that each extra day after commencing chemotherapy before the development of a grade 1 toxicity resulted in a lower risk of subsequently developing a grade 2 or worse toxicity in the case of PPE (hazard ratio (HR) 0.99, 95% confidence interval (CI) 0.985–0.994; *P*<0.001) and stomatitis (HR 0.965, 95% CI 0.95–0.98; *P*<0.001). This result suggested that the time at which grade 1 toxicity initially developed could be used to identify patients who were at higher risk of subsequently developing a more severe toxicity.

The median time until the first development of any grade 1 toxicity was 28 days. Patients with early grade 1 toxicities were therefore defined as those patients who developed grade 1 but not grade 2 or worse diarrhoea, PPE or stomatitis before completing 28 days of chemotherapy. This time point also predates the times at which the largest proportions of patients have grade 2 or worse toxicities (see Figure 1B-3B).

In all, 55.1% of patients with early grade 1 PPE subsequently developed grade 2 or worse PPE compared with 27.3% of patients without early grade 1 PPE (*P*<0.001). A total of 19.9% of patients with early grade 1 diarrhoea subsequently developed grade 2 or worse diarrhoea compared with 11.9% of patients without early grade 1 diarrhoea (*P*=0.003). Totally, 19.5% of patients with early grade 1 stomatitis subsequently developed grade 2 or worse stomatitis compared with 11.4% of patients without early grade 1 stomatitis (*P*=0.003).

### Multivariate Cox regression analysis of baseline and temporal factors associated with toxicities

The significance of early grade 1 toxicity in addition to other clinical and laboratory parameters as prognostic factors associated with the development of more severe toxicity was examined using multivariate Cox regression analysis (see Table 3

**Table 3:** Multivariate Cox regression analysis of factors predicting toxicity with PVI 5FU

		**Multivariate Cox regression**				
**Factor**	**Groups**	***N***	***P***	**HR**		**95% CI of HR**
*Diarrhoea ⩾grade 2*						
Early toxicity	Early diarrhoea	196	0.008	1.541	1.121	2.119
	No early diarrhoea	1172		1		
						
*PPE ⩾grade 2*						
Performance status	PS 0-1	1033	<0.001	1.692	1.302	2.199
	PS 2-4	335		1		
Sex	Female	592	<0.001	1.413	1.173	1.700
	Male	776		1		
Bilirubin	Low <12 *μ*mol l^−1^	716		1		
	High ⩾12 *μ*mol l^−1^	652	0.001	1.357	1.127	1.635
Early toxicity	Early PPE	218	<0.001	2.450	1.992	3.012
	No early PPE	1150		1		
	Early diarrhoea	196	0.001	1.483	1.167	1.886
	No early diarrhoea	1172		1		
						
*Stomatitis ⩾grade 2*						
Sex	Female	593	0.002	1.403	1.135	1.733
	Male	776		1		
Age	Continuous	1369	0.002	1.016	1.006	1.026
ALT	Low <16 U l^−1^	683	0.002	1		
	High ⩾16 U l^−1^	686		1.390	1.126	1.717
Urea	Low <4.8 mmol l^−1^	705	0.002	1		
	High ⩾4.8 mmol l^−1^	664		1.422	1.143	1.769
Early toxicity	Early PPE	218	<0.001	1.574	1.234	2.009
	No early PPE	1151		1		

). Early grade 1 diarrhoea predicted grade 2 or worse diarrhoea. Female sex, good performance status, elevated bilirubin, early grade 1 PPE and early grade 1 diarrhoea were independent prognostic factors for the development of grade 2 or worse PPE. Female sex, increased age, ALT, urea and early grade 1 PPE were independent prognostic factors for the development of grade 2 or worse stomatitis. No other factors including primary tumour site and chemotherapy arm were significantly associated with toxicity.

### Identification of patients at high risk of toxicity

The multivariate Cox regression analysis allows identification of subgroups of patients at higher risk of toxicity with PVI 5FU. Based on the multivariate Cox regression analysis, older, female patients with good performance status, impaired liver function (alanine transaminase; ALT⩾16 U l^−1^ or bilirubin⩾12 *μ*mol l^−1^) and renal function (urea⩾4.8 mmol/l^−1^) who develop early grade 1 PPE alone or in combination with diarrhoea could be considered for early dose reduction, at the time of development of early toxicity, as this group of patients is at the highest risk of grade 2 or worse PPE and stomatitis.

## DISCUSSION

This study represents the largest analysis of the toxicities associated with infused 5FU and provides information about the development of toxicities with time and evaluation of factors predicting the development of moderate and severe toxicity. A previous meta-anlysis of five individual trials assessed factors predicting nonhaematological toxicities in patients receiving infused 5FU (Meta-analysis Group In Cancer, 1998b). However, this study is limited as only 607 patients receiving infused 5FU were included in the analysis, patients received a variety of different schedules of infused 5FU and no data were provided relating to the time course of toxicities.

This type of analysis is important as the more tolerable toxicity profile of infused 5FU has led to greater interest in the use of this schedule of administration. In advanced colorectal cancer, there have been concerns relating to increased rates of early mortality using bolus schedules of 5FU with irinotecan, which were not observed using infused schedules (Douillard *et al*, 2000; Rothenberg *et al*, 2001). In addition, for adjuvant treatment of colorectal cancer, there is increasing recognition that infused schedules of 5FU are as effective as bolus schedules, but with a lower incidence of toxicity (Andre *et al*, 2002). PVI 5FU is also commonly used as a component of treatment of advanced oesophago-gastric cancer (Webb *et al*, 1997).

### Baseline clinical factors associated with toxicity

Our analysis provides data relating to baseline clinical and laboratory factors, which are associated with a greater risk of developing moderate-to-severe toxicities. These include female sex, older age and elevated ALT and urea for stomatitis and female sex, better performance status and elevated bilirubin for PPE.

A previous analysis has suggested that good performance status predicts superior response rates and more prolonged survival in advanced colorectal cancer (Assersohn *et al*, 1999). Consequently, factors such as better performance status are likely to indicate those patients who are likely to have more prolonged progression-free survival and who may therefore receive chemotherapy for more protracted periods of time (Borner *et al*, 1998). The association of better performance status with the development of PPE has also been noted by other investigators (Meta-analysis Group In Cancer, 1998b). However, the aim of this analysis was to identify factors, which could be identified early during treatment, which were independently associated with toxicity rather than survival or response. Any decision about whether to use chemotherapy inevitably involves a balanced judgement about both the likely benefits of treatment as well as the risk of toxicity.

Other factors, such as female sex and age, are similar to those found by other investigators who have demonstrated that bolus schedules of 5FU caused more severe leucopenia in older female patients and more severe stomatitis in older patients (Zalcberg *et al*, 1998; Popescu *et al*, 1999). A recent analysis also demonstrated that female patients suffered more severe toxicity using bolus schedules of 5FU (Sloan *et al*, 2002). Older age and female sex have also been previously associated with PPE using infused 5FU (Meta-analysis Group In Cancer, 1998b).

The increased toxicity associated with 5FU administration in older and female patients may reflect altered catabolism or clearance of 5FU in these patients. More than 80% of an administered dose of 5FU is eliminated by catabolism by the enzyme dihydropyrimidine dehydrogenase (DPD). Severe 5FU toxicity may occur in patients with partial or complete DPD deficiency and these individuals show marked impairment of 5FU clearance (Diasio *et al*, 1988; Lu *et al*, 1993; Milano *et al*, 1999). Fluorouracil clearance has been shown to be impaired in females although it does not appear to be affected by age (Milano *et al*, 1992).

### Prediction of toxicity using DPD levels

On this basis, measurement of DPD levels has been suggested as a possible method to identify patients at increased risk of 5FU toxicity. However, there are limitations with such an approach. Although the majority of 5FU is metabolised by the liver, assessment of DPD levels is typically based on the levels of the enzyme expressed in peripheral blood mononuclear cells because of their easier accessibility. Unfortunately, assessment of peripheral blood DPD levels correlates only weakly with 5FU clearance and is a poor predictor of 5FU toxicity (Fleming *et al*, 1992; Etienne *et al*, 1994). Even among patients who had developed severe 5FU toxicities, who might have been expected to be relatively DPD deficient, only a proportion of patients had reduced peripheral blood DPD levels (Milano *et al*, 1999; van Kuilenburg *et al*, 2000). Therefore, measurement of DPD levels before 5FU treatment has limited utility for the prediction of patients destined to develop severe 5FU-related toxicities. This limitation may arise because assessment of DPD levels in this fashion does not accurately reflect the body's capacity to metabolise 5FU or because other factors may also predispose to 5FU toxicity.

### Prediction of toxicity using pharmacokinetic monitoring

An alternative approach to predict and ultimately prevent severe 5FU toxicity is pharmacokinetic monitoring. Patients with DPD deficiency may demonstrate a markedly altered pharmacokinetic profile with significant reduction in the plasma levels of 5FU metabolites. The impaired clearance of 5FU is thought to contribute to its toxicity in these patients. Pharmacokinetic evaluation provides a more direct information about whole-body 5FU catabolism, and small studies suggest that with infused 5FU, higher plasma levels of 5FU correlate with increased toxicity (Findlay *et al*, 1996; Gamelin *et al*, 1996). However, plasma 5FU levels have been found to show wide inter- and intrapatient variation and larger studies have failed to show any relation between steady-state plasma 5FU levels and 5FU toxicity (Jodrell *et al*, 2001). Thus, 5FU pharmacokinetic assessment is not routinely used for the prediction of patients at increased risk of toxicity.

### Use of temporal factors in combination with baseline clinical factors to assess risk of toxicity

This study provides a strong empirical basis for the use of information about the temporal development of toxicity in combination with baseline clinical factors in order to assess the risk of toxicity. While it appears logical that patients who develop early grade 1 toxicity would be at higher risk of developing a subsequent more severe toxicity, the extent of the risk and the possible implications for patient management have never previously been evaluated. Thus, the data suggest that older female patients with good performance status and impaired liver function who develop early grade 1 PPE or the combination of early grade 1 PPE and diarrhoea would be at a higher risk of grade 2 or worse stomatitis and PPE. Such patients could receive a reduction in the dose of 5FU at the time of development of early grade 1 toxicity, which could prevent the development of more severe toxicity altogether. This approach, if successful, would be likely to achieve significant benefit in improvements in quality of life as well as minimise treatment interruption due to toxicity.

However, in order to evaluate rigorously such an approach, it will be important to validate the models generated in this study prospectively. It will also be important to study the use of early dose reduction for selected patients at high risk of toxicity prospectively. This should involve assessment of response, progression-free survival, overall survival and quality of life in addition to toxicity outcomes in order to determine whether efficacy and palliative benefit is affected using such an approach.

### Future applications

An increased understanding of factors associated with toxicity due to chemotherapy may potentially allow early identification of patients at risk of severe toxicity. This study suggests that evaluation of the temporal pattern of toxicity can assist in this process. Similar approaches using data derived from large patient cohorts treated with other chemotherapy agents may allow identification of prognostic factors, including temporal factors associated with toxicity due to other drugs, which could prove useful for clinical management.
